# Temporal variability in pupillary asymmetry reflects ADHD-related traits in preschool and early school-aged children

**DOI:** 10.3389/fcogn.2026.1781233

**Published:** 2026-03-11

**Authors:** Sou Nobukawa, Isshu Wakita, Aya Shirama, Shingo Ofuchi, Ayumu Ueno, Kohei Okamoto, Hina Nishimura, Masahide Seto, Ayaka Yamauchi, Shuji Igawa, Tomiki Sumiyoshi

**Affiliations:** 1 Department of Computer Science, Chiba Institute of Technology, Narashino, Chiba, Japan; 2 Graduate School of Information and Computer Science, Chiba Institute of Technology, Narashino, Chiba, Japan; 3 Research Center for Mathematical Engineering, Chiba Institute of Technology, Narashino, Chiba, Japan; 4 Department of Preventive Intervention of Psychiatric Disorders, National Institute of Mental Health National Center of Neurology and Psychiatry, Kodaira/Tokyo, Japan; 5 Graduate School of Data Science, Nagoya City University, Nagoya, Aichi, Japan; 6 Innovation Center, SMK Corporation, Tokyo, Japan; 7 Japan Health Research Promotion Bureau, Tokyo, Japan

**Keywords:** attention-deficit/hyperactivity disorder, functional asymmetry, interocular asymmetry, preschool- and early school-aged children, pupillometry

## Abstract

Attention-deficit/hyperactivity disorder (ADHD) often emerges in early childhood; however, objective validated biomarkers for its early detection remain limited. In this study, we aimed to identify pupil-based candidate physiological markers (i.e., physiological correlates) associated with ADHD-related traits in preschool- and early school-aged children. To this end, we recorded the pupil diameters of typically developing children without an ADHD diagnosis during a simple fixation task. From these data, we extracted multiple features, including the mean pupil size, temporal variability, and interocular asymmetry in both magnitude and variability. ADHD-related tendencies were assessed using the ADHD Rating Scale-5. Among the features analyzed, only the temporal variability of interocular asymmetry (VarLRdiff) showed a significant positive correlation with ADHD Rating Scale-5 total and subscale scores. This association likely reflects the combined effects of immature autonomic regulation and functional asymmetry in the neural circuits involving the locus coeruleus. In contrast, other pupil-based indices showed no significant correlations, potentially because of developmental ceiling effects and the short evaluation duration. These findings suggest that VarLRdiff may serve as a promising candidate physiological marker of ADHD-related traits in young children, requiring further validation. Future studies incorporating longer recordings, advanced analytical methods, and evaluation of test–retest stability, as well as longitudinal follow-up, are warranted to evaluate its potential utility for early screening, pending clinical and longitudinal validation.

## 1 Introduction

Attention-deficit/hyperactivity disorder (ADHD) is a neurodevelopmental condition characterized by difficulties in attention regulation and behavioral inhibition during childhood and is known to exert widespread impacts on learning, interpersonal relationships, and even the broader family environment (Coghill and Seth, 2015; Faraone et al., 2021). In particular, the importance of screening during the preschool years has been widely recognized, as symptoms of ADHD can emerge as early as before the age of 5 years (Wolraich et al., 2011). Moreover, ADHD-related traits have been reported to be continuously distributed even among children with typical development who do not meet diagnostic criteria, suggesting that identifying these tendencies at an early developmental stage is critical for effective screening (Balázs and Keresztény, 2014; McLennan, 2016). However, current diagnostic approaches, which are primarily based on interviews and behavioral reports, are subject to variability in accuracy due to multiple influencing factors, including sex, age, race, socioeconomic status, symptom severity, presence of comorbidities, and social, cultural, and genetic factors related to the age of onset and diagnosis (Socanski et al., 2013; Rocco et al., 2021; Musullulu, 2025). Given these limitations, developing candidate neurophysiological markers or physiological correlates that may support the objective assessment of ADHD-related tendencies in typically developing children at the preschool stage is imperative (Chen et al., 2023).

Hyperactivity, impulsivity, and inattention, the core symptoms of ADHD, are associated with multiple neural systems (Curatolo et al., 2010; Mehta et al., 2019; MacDonald et al., 2024). Among the most prominent are the dopaminergic system (MacDonald et al., 2024), the locus coeruleus–noradrenaline (LC-NA) system (Maness et al., 2022), and the prefrontal cortex, which receives projections from these systems (Curatolo et al., 2010). To investigate the neural activity underlying these systems, various neuroimaging modalities, including functional magnetic resonance imaging (fMRI), magnetoencephalography (MEG), near-infrared spectroscopy, MRI, and electroencephalography (EEG), have been extensively employed (Chen et al., 2023). In particular, fMRI and MRI provide high spatial resolution for assessing anatomical and hemodynamic changes in deep brain structures, whereas EEG and MEG enable analyses focusing on specific frequency bands and functional networks directly related to attentional processes (Lenartowicz and Loo, 2014; Zhan et al., 2017; Dor-Ziderman et al., 2021; Slater et al., 2022; Chen et al., 2021, 2023). In parallel, increasing attention has been paid to physiological indices that indirectly reflect neural activity, such as pupil diameter and heart rate variability (Idrees et al., 2023). Among these, the pupil diameter serves as a surrogate marker for LC-NA activity, which regulates arousal and attentional states (Aston-Jones and Cohen, 2005). The LC is situated at the central origin of the sympathetic nervous system, which mediates pupil dilation via noradrenergic transmission, and the parasympathetic nervous system, which controls constriction via cholinergic pathways. Accordingly, LC activity can be inferred by monitoring pupillary responses. Moreover, pupil diameter fluctuations include spontaneous oscillatory dynamics, known as hippus (Usui and Stark, 1982), which are thought to reflect intrinsic LC activity that is difficult to detect using EEG or MEG (Nobukawa et al., 2024). Pupil-based signals also encompass a broader time-frequency domain < 10 Hz (Bouma and Baghuis, 1971; McLaren et al., 1992; Ukai et al., 1997) than the typical range captured by fMRI, offering rich information about neuromodulatory fluctuations.

Recent studies utilizing eye-tracking techniques have begun to explore the relationship between pupil-linked neuromodulatory dynamics and ADHD symptomatology (Levantini et al., 2020; Chen et al., 2023). These investigations focused primarily on group-level comparisons between individuals diagnosed with ADHD and typically developing (TD) controls (Wainstein et al., 2017; Shirama et al., 2020). For example, during cognitive tasks, ADHD groups often show attenuated phasic pupil responses (Wainstein et al., 2017; Shirama et al., 2020), whereas under resting-state conditions, enlarged tonic pupil diameters, interpreted as signs of LC hyperactivity, have been observed (Nobukawa et al., 2021a). Beyond static metrics such as the average pupil size, the temporal dynamics of spontaneous pupil fluctuations (Artoni et al., 2019) also appear to differ between groups, with decreased signal complexity reported in ADHD populations (Nobukawa et al., 2021a). In addition, asymmetries in LC activity have been suggested, manifesting as lateralized differences in pupil diameter and fluctuation patterns (Nobukawa et al., 2021a; Kumano et al., 2022). Despite these advances, most prior studies were limited to adults or adolescents, with little attention paid to younger populations, such as preschool-aged children (Chen et al., 2023). Moreover, nearly all of these studies adopted a categorical design, contrasting the ADHD and TD groups (Chen et al., 2023). Limited studies have examined the relationship between ADHD-related traits and ocular indices in TD populations, where such traits are understood to be continuously distributed (Sonuga-Barke and Halperin, 2010). One notable exception is a study by Poynter, which found associations between pupil size asymmetry and attentional traits in TD participants ranging in age from 18 to 40 years old (Poynter, 2017). This implies that pupil size asymmetry is a promising candidate feature for capturing continuously varying ADHD-related traits within the TD population, consistent with the view that ADHD is increasingly conceptualized as a dimensional construct rather than a purely categorical disorder (Sonuga-Barke and Halperin, 2010). However, to the best of our knowledge, no studies have addressed whether continuously varying ADHD tendencies in TD children are reflected spontaneously in pupils. Clarifying these associations will provide valuable insights into the neurophysiological underpinnings of attentional traits and contribute to the development of objective early-stage screening tools.

Among the various pupil-based indices, interocular asymmetry in pupil size and temporal variability are particularly noteworthy. Previous computational modeling studies have suggested that these features may reflect the functional asymmetry and dynamics of neural activity within the LC system (Kumano et al., 2022). In the present study, we aimed to explore whether a set of pupil asymmetries and their temporal variability are associated with ADHD-related traits and could collectively serve as informative physiological indices for capturing attentional tendencies in typically developing children. This exploratory, hypothesis-generating approach was theoretically motivated by prior findings on pupil asymmetry and autonomic regulation, and was extended here to preschool and early school-aged children, a population that has received little attention in ocular biomarker research. The findings are intended to inform future confirmatory studies and the validation of candidate neurophysiological markers. For this purpose, we measured the pupil diameter using an eye tracker during a simple fixation task in TD children without an ADHD diagnosis. From the resulting time-series data, we extracted multiple features including interocular asymmetry in the left and right pupil diameters and their temporal variability. These features were then statistically analyzed in relation to ADHD tendencies, which were quantitatively assessed using the ADHD Rating Scale-5 (ADHD-RS-5) through correlation analysis.

## 2 Materials and methods

### 2.1 Participants

This study included 59 participants. Exclusion criteria were assessed using a parent-report screening questionnaire administered prior to the experiment, and children with previously diagnosed neurological or developmental disorders were excluded. The physical characteristics of the participants are summarized in Table 1. *A priori* power analysis was conducted using the statsmodels package in Python to estimate the sample size required for correlation detection. Assuming a medium effect size (correlation coefficient *r* = 0.5) (Poynter, 2017), an alpha level of 0.05, and statistical power of 0.80 (two-tailed test), the analysis indicated that a minimum of 47 participants would be required to detect a statistically significant correlation. The Japanese version of the ADHD-RS-5 (DuPaul et al., 2016) was completed by the participants' caregivers. The ADHD-RS-5 is a parent- or teacher-reported questionnaire that incorporates the 18 symptoms of ADHD outlined in the fifth edition of the Diagnostic and Statistical Manual of Mental Disorders (American Psychiatric Association, 2013). It was developed to assess the severity of ADHD symptoms and evaluate treatment in children aged 5–18. The raw scores were converted into percentile scores, indicating the relative position of each participant's score within a normative distribution. These percentiles represent the percentage of individuals in the normative sample, stratified by sex and the 5–7-year age group, who scored below the participant's score. The ADHD-RS-5 is designed for children aged 5–18 years and was administered in its standard form in the present study as a continuous measure of ADHD-related traits rather than for diagnostic classification, with the same scoring procedure applied across both preschool and early school-aged children. All of the participants had normal vision and did not wear glasses or contact lenses. All participants were enrolled in age-appropriate educational programs and showed no signs of intellectual difficulty, based on their caregiver reports. On the day of the experiment, the participants did not ingest caffeine, nicotine, or medications that could affect eye movement or pupil diameter. Participants were recruited through a commercial recruitment agency and internal announcements within the SMK Corporation. All measurements were conducted at the facilities of the SMK Corporation. All procedures adhered to the Declaration of Helsinki, and the study protocol was reviewed and approved by the Ethics Committee of the Chiba Institute of Technology (approval number 2024-05-01). Caregivers provided written informed consent for participation in the study.

**Table 1:** Physical characteristics in participants.

**Items**	**Values**
Age (month)	60–96 (mean 76.7, SD 10.3)
Male/female	24/35
**ADHD-RS-5 (%tile)**
Total score	1–100 (mean 48.3, SD 26.9)
IN score	1–95 (mean 30.8, SD 26.7)
Hyp/I score	1–98 (mean 40.0, SD 27.4)

%tile, percentile score; ADHD-RS-5, attention-deficit/hyperactivity disorder rating scale-5; SD, standard deviation.

### 2.2 Experimental procedure and pupil diameter recording

**Figure 1:**
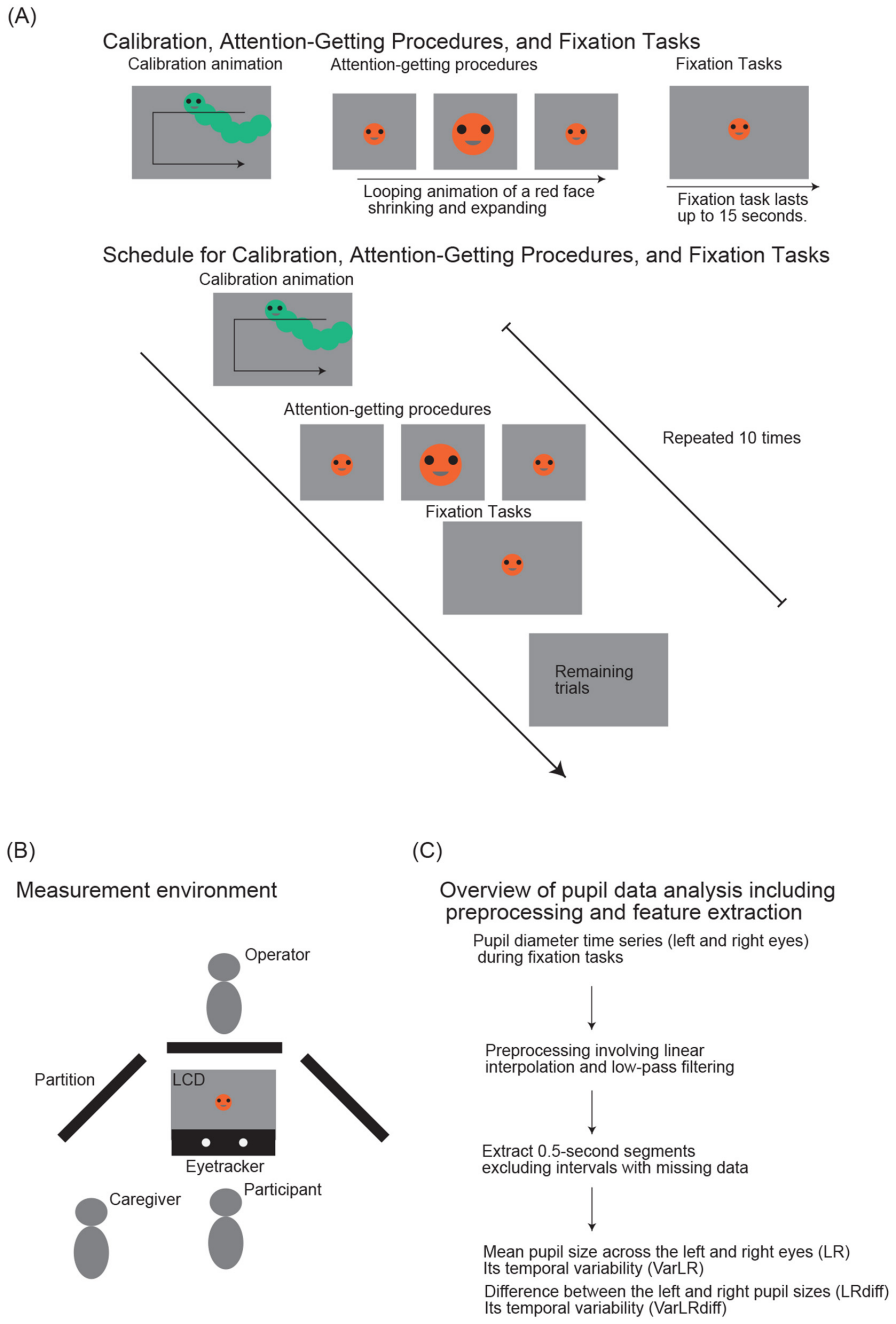
Conceptual overview of the experimental procedure, measurement setup, and pupil data analysis pipeline. **(A)** Sequence of calibration, attention-getting procedures, and fixation tasks. Stimuli were presented in a repeated schedule, with each fixation trial lasting up to 15 s. Attention-getting animations were used to reorient gaze when necessary. **(B)** Measurement environment showing the positions of the participant (child), the eye tracker, and the caregiver seated next to the participant. **(C)** Pupil data analysis pipeline, including preprocessing steps (blink detection, interpolation, normalization) and subsequent feature extraction.

Figure 1 presents a conceptual overview of the experimental procedure (Figure 1A) and the measurement setup (Figure 1B). In the visual task illustrated in Figure 1A, a caterpillar animation designed for infants was presented as an attention-getter. The animation moved across the screen while repeatedly expanding and contracting and was used for pupil calibration. Subsequently, another attention-getting animation featuring a red smiling face that expanded and contracted was displayed to guide the participant's gaze toward the center of the screen. The experimenter monitored the participant's gaze in real-time using an eye tracker. Once the gaze was confirmed to be centered, the stimulus was switched to a static red smiling face (fixation image) with an RGB value of (*R* = 255, *G* = 0, *B* = 0) presented on a gray background (*R* = 130, *G* = 130, *B* = 130). The fixation image was displayed for up to 15 s. If the participant's gaze deviated from the center or if 15 s passed, the stimulus was terminated, and the remaining number of trials was shown before representing the attention-getter. This cycle of attention-getter and fixation image presentation was repeated up to 10 times. If the participant did not attend to the visual stimuli, the caregiver was seated next to the participant and the operator gently encouraged them to look at the screen. The pupil diameter was recorded while the participants sat in front of a monitor subtending 49.4° × 29.0° of visual angle at a viewing distance of approximately 65 cm in a well-lit room (illuminance range: 362 − 612 lx; mean: 527.7 lx; SD: 48.5 lx). The fixation image subtended 2.64° × 2.64° of visual angle, with luminance ranging from 24.1 to 47.4 cd/m^2^ (mean: 33.1 cd/m^2^; SD: 3.30 cd/m^2^).

To minimize head and neck movements in a child-friendly manner, the participants were instructed to sit fully against the chair's backrest, which allowed for a natural, non-intrusive posture that was well tolerated by the children. To ensure that the participants were relaxed and comfortable before the experiment, they were allowed to spend approximately 5 min in a play area adjacent to the experimental room. The experiment began only after the participants, their caregivers, and the experimenter agreed that they were ready to start. In addition, to avoid excessive fatigue, all measurements were conducted between 9:30 a.m. and 4:30 p.m. Visual stimuli were developed and presented using Tobii Pro Lab Full Edition (version 24.21) on a Windows 10 operating system. Eye movement and pupil data were recorded using a Tobii Pro Fusion eye tracker at a sampling rate of 120 Hz.

### 2.3 Analysis of pupil diameter time series and evaluation metrics

The following preprocessing steps were applied to the pupil-diameter time series, as illustrated in Figure 1C. First, missing values, primarily caused by eyeblinks, were linearly interpolated. Second, a low-pass filter with a cutoff frequency of 20 Hz was applied to retain the main frequency components of pupil dynamics (Bouma and Baghuis, 1971; McLaren et al., 1992; Ukai et al., 1997). The preprocessed time series were then segmented into 0.5-s epochs.

Epochs containing ≥20 contiguous missing samples (i.e., 20 consecutive time-steps at 120 Hz = 0.166 s) were excluded from further analysis. This threshold corresponds to approximately 16.6% missing data within a 0.5-s epoch. Previous pupillometry studies evaluating dynamic characteristics using complexity measures (Nobukawa et al., 2021b) adopted stricter exclusion criteria, such as rejecting epochs with >10% missing data. Because the present study focused on asymmetry variability based on temporal averages and variability—rather than dynamic characteristics—we employed a slightly more lenient criterion. This allowed us to retain a sufficient number of usable epochs while avoiding the influence of extended unmeasurable periods caused by blinks or eye movements. The distribution of the number of valid epochs across participants is reported in the Supplementary material.

As an evaluation metric, we calculated the temporal average of the mean pupil size across the left and right eyes within each epoch, referred to as LR. We also calculated the temporal average of the difference between the left and right pupil sizes within each epoch (i.e., left minus right), referred to as LRdiff (Poynter, 2017; Kumano et al., 2022): These values were then averaged across all valid epochs for each participant. To assess temporal variability, we calculated the standard deviations of the mean pupil size (VarLR) and LRdiff (VarLRdiff) across epochs. In our previous studies, which focused on the temporal variability of pupil size and interocular differences, we used more complex indices, such as transfer entropy and differences in sample entropy (Nobukawa et al., 2021a; Kumano et al., 2022). However, given the limited recording duration feasible for preschool and early school-aged children in the current study, we employed VarLR and VarLRdiff as simpler yet effective metrics for capturing variability, making them suitable for short-duration recordings.

### 2.4 Statistical analysis

We used Spearman's rank correlation coefficient (ρ) to evaluate the relationship between ADHD symptom severity, as measured by ADHD-RS-5 scores, and four pupil-based indices (LR, VarLR, LRdiff, and VarLRdiff). Normality of these indices was assessed using the Kolmogorov–Smirnov test, and because VarLRdiff did not follow a normal distribution, nonparametric correlation analyses were adopted (see Supplementary material). In the primary analysis, we examined the correlations between the total ADHD-RS-5 score and the four indices by applying the Benjamini–Hochberg false discovery rate (FDR) correction (*q* < 0.05) for these comparisons. Only the indices that survived FDR correction were subjected to secondary analyses involving the ADHD-RS-5 subscale scores: hyperactivity/impulsivity score (ADHD-RS-5 Hyp/I) and inattentiveness score (ADHD-RS-5 IN). The same FDR correction (*q* < 0.05) was applied to the secondary analysis.

## 3 Results

**Figure 2:**
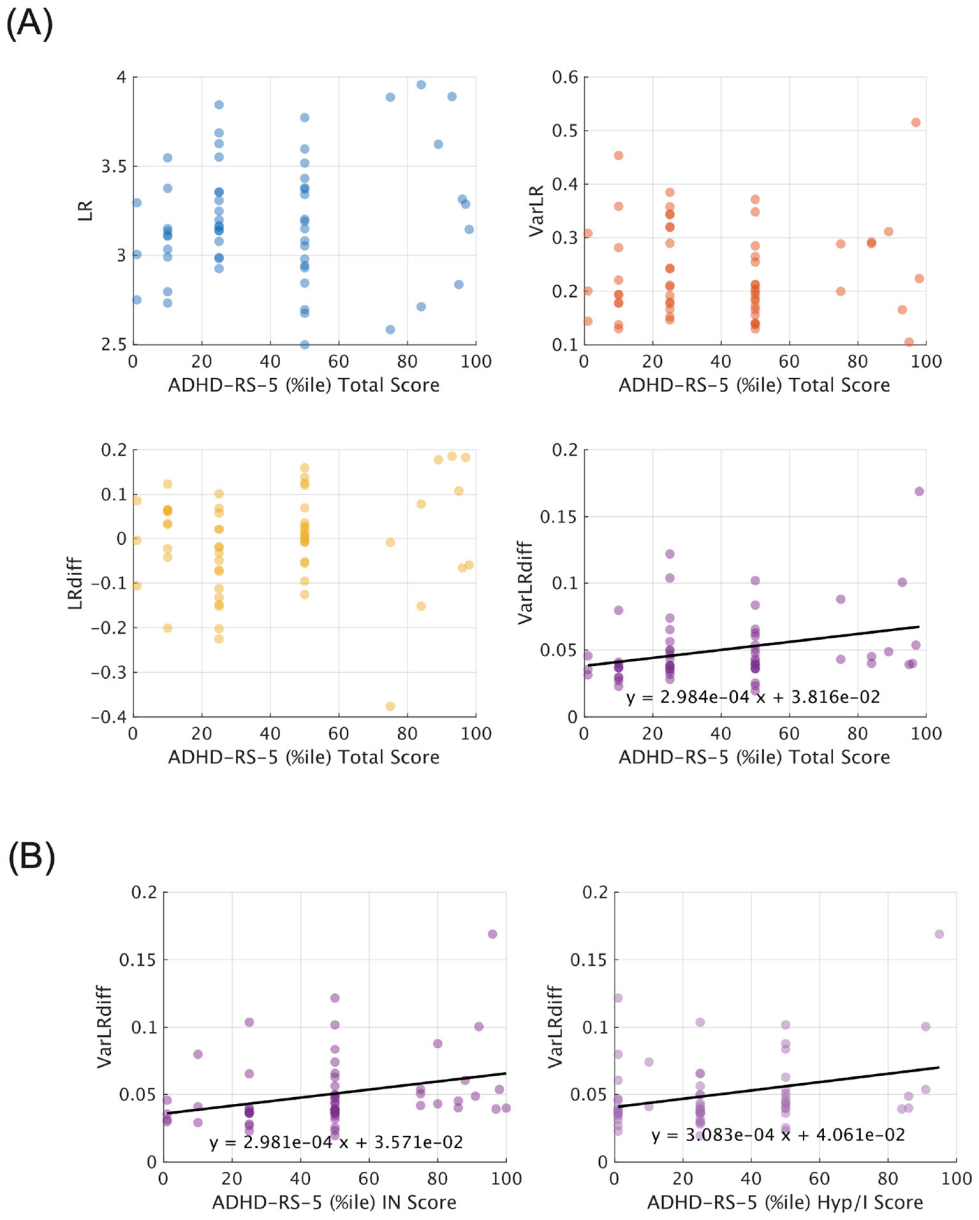
Scatter plots showing the associations between ADHD-RS-5 percentile scores (total, IN, and Hyp/I) and pupil-based indices (LR, VarLR, LRdiff, and VarLRdiff). **(A)** Scatter plots between the ADHD-RS-5 total percentile score and pupil indices (LR, VarLR, LRdiff, and VarLRdiff). In the VarLRdiff panel, where a significant strong correlation with the total score is identified (*q* < 0.05) (Table 2), the regression line is shown. **(B)** Scatter plots between the ADHD-RS-5 subscale scores (IN and Hyp/I) and VarLRdiff, which showed a significant strong correlation with the total score. Regression lines are displayed in both panels, as significant strong correlations are also confirmed for both subscale scores (*q* < 0.05) (Table 2). Regression lines from simple linear regression analyses are shown for illustrative purposes, and the regression coefficients were not standardized.

Table 2 presents the Spearman's rank correlation coefficients (ρ) and *p*-values for the associations between the ADHD-RS-5 total percentile score and the pupil-based indices (LR, VarLR, LRdiff, and VarLRdiff). Among these indices, only VarLRdiff exhibited a strong positive correlation (*q* < 0.05). Subsequent correlation analyses between VarLRdiff and ADHD-RS-5 subscale percentile scores—IN and Hyp/I—also revealed strong positive correlations (*q* < 0.05). Scatter plots corresponding to these correlation analyses are shown in Figure 2. The same pattern of associations was also confirmed when VarLRdiff was computed without applying linear interpolation to the pupil time series (see Supplementary material).

**Table 2:** Spearman's rank correlation coefficients (ρ) and *p*-values for associations between ADHD-RS-5 percentile scores (total, IN, and Hyp/I) and pupil indices (LR, VarLR, LRdiff, and VarLRdiff).

**Items**	**LR**	**VarLR**	**LRdiff**	**VarLRdiff**
Total score	ρ = 0.12 (*p* = 0.351)	ρ = 0.01 (*p* = 0.942)	ρ = 0.12 (*p* = 0.365)	**ρ = 0.35** (***p* = 0.007**)
IN score	–	–	–	**ρ = 0.40** (***p* = 0.002**)
Hyp/I score	–	–	–	**ρ = 0.29** (***p* = 0.026**)

Subscale correlations were tested only if the total score showed a significant association after FDR correction (*q* < 0.05). “–” indicates that no test was performed. For VarLRdiff, strong positive correlations were observed with the total, IN, and Hyp/I scores (*q* < 0.05). Bold values indicate results that remained significant after FDR correction (*q* < 0.05).

As age and sex are potential confounding factors for pupil-based indices, we further examined their influence on VarLRdiff. Spearman's rank correlation analysis revealed no association between VarLRdiff and age (in months) (ρ < 0.001, *p* = 1.0). In addition, no significant differences were observed between males and females (*t* = 1.485, *p* = 0.146). Therefore, the strong correlations observed between VarLRdiff and the ADHD-RS-5 scores did not appear to be attributable to age or sex.

Furthermore, we examined whether ADHD-RS-5 scores themselves were associated with age or sex. Spearman's rank correlation analyses revealed no significant associations between age (in months) and the ADHD-RS-5 total and subscale percentile scores (total: ρ = 0.12, *p* = 0.36; IN: ρ = 0.15, *p* = 0.25; Hyp/I: ρ = −0.01, *p* = 0.96). Mann–Whitney *U*-tests also indicated no significant sex differences in these scores (total: *U* = 661.0, *p* = 0.35; IN: *U* = 660.0, *p* = 0.34; Hyp/I: *U* = 670.5, *p* = 0.43). These findings indicate that ADHD-RS-5 ratings were independent of age and sex in our sample, supporting the interpretation that the observed associations between VarLRdiff and ADHD-related traits were not confounded by these demographic factors.

## 4 Discussion and conclusion

In this study, we aimed to identify ADHD-related traits in preschool and early school-aged children by analyzing multiple features derived from pupil diameter, including pupil size (LR), temporal variability (VarLR), interocular asymmetry (LRdiff) between the left and right pupils, and temporal variability (VarLRdiff). Correlation analyses revealed that VarLRdiff showed a significant and strong positive correlation with the total ADHD-RS-5 score and with both subscale scores: IN and hyperactivity/impulsivity (Hyp/I).

First, understanding why LRdiff did not show a significant correlation with ADHD-related traits, which is inconsistent with the previous findings in adults is necessary (Poynter, 2017). Children in the early developmental stages exhibit higher levels of activity in both the sympathetic and parasympathetic nervous systems than adults (Bufo et al., 2022). Consequently, pupil dynamics in children are characterized by greater variability, owing to the stronger influence of these autonomic mechanisms (Winston et al., 2020; Bufo et al., 2022). Consequently, LRdiff, which represents the temporal average of interocular asymmetry, may have been obscured by this high level of variability during the evaluation period. This likely reduces its effectiveness as a metric for capturing ADHD-related traits in early developmental stages.

We must also consider why VarLRdiff, unlike LRdiff, was significantly correlated with ADHD-related traits. The asymmetry in the pupil size between the left and right eyes can be attributed to two main factors. One factor is the imbalance in both neural structure and activity between the left and right hemispheres, which has been reported in individuals with ADHD (Doi and Shinohara, 2017; Douglas et al., 2018; He et al., 2022). In particular, asymmetry in the activity of the left and right LC, which plays a central role in pupil size regulation, is associated with ADHD pathology and traits (Poynter, 2017; Kumano et al., 2022). Another contributing factor is the neural pathways that control the pupil size. The LC projects bilaterally and inhibits both the ipsilateral and contralateral regions of the Edinger–Westphal nucleus, which controls the pupil sphincter muscles (Liu et al., 2017). Our previous research suggested that contralateral projections from the LC to the Edinger–Westphal nucleus, which are part of the parasympathetic neural pathway, play a critical role in generating interocular asymmetry of pupil size, depending on the LC activity levels (Nobukawa et al., 2021b). Collectively, in the early developmental stages, when the immaturity of the autonomic nervous system leads to greater neural variability (Winston et al., 2020; Bufo et al., 2022)—interocular pupil asymmetry may emerge intermittently, driven by underlying hemispheric neural imbalances, and become more pronounced in association with ADHD traits. This effect may be amplified in children with strong ADHD traits whose autonomic regulation, involving both the sympathetic and parasympathetic nervous systems, is known to be unstable (Castro Ribeiro et al., 2024). The combination of these factors likely contributes to the increased variability in interocular asymmetry (VarLRdiff) observed in children with ADHD-related traits. Because the present study employed a brief fixation task with minimal cognitive demands, the observed pupil dynamics are likely to reflect baseline or tonic modes of locus coeruleus activity rather than task-evoked phasic responses (Shirama et al., 2020; Nobukawa et al., 2021a). In this sense, VarLRdiff may capture relatively stable, trait-like aspects of neuromodulatory and autonomic regulation, rather than transient state-dependent effects.

Additionally, understanding why LR (mean pupil size) and VarLR (temporal variability of pupil size) did not show significant correlations with ADHD-related traits is crucial, despite previous findings in adults indicating that larger pupil size and reduced temporal variability, often referred to as reduced complexity, are associated with ADHD pathology (Shirama et al., 2020; Nobukawa et al., 2021a). In early developmental stages, children generally exhibit larger pupil sizes than adults (Bufo et al., 2022), which may have led to a ceiling effect, thereby diminishing the sensitivity of LR in detecting ADHD-related tendencies. Regarding VarLR, prior studies have shown that the temporal variability associated with ADHD pathology is best captured using complex dynamic metrics such as sample entropy (Nobukawa et al., 2021a). However, owing to the short evaluation duration in this study—necessitated by the challenges of conducting experiments with children in the early developmental stages—such advanced metrics could not be applied. Instead, we used temporal standard deviation to estimate variability, but this simple measure may not have been sensitive enough to detect the subtle temporal dynamics of pupillary behavior associated with ADHD. The VarLR did not significantly correlate with ADHD-related traits in this study.

At the same time, alternative explanations should be considered when interpreting the observed association between VarLRdiff and ADHD-related traits. In the context of pupillometry, variability-based measures are particularly sensitive to measurement-related factors such as blink events, brief gaze deviations, partial eyelid occlusion, and head movements, all of which can introduce artefactual fluctuations in pupil diameter estimates (Hayes and Petrov, 2016; Hooge et al., 2021). These effects may be further amplified in developmental samples, including preschool-aged children, due to increased motor activity and reduced fixation stability. These alternative explanations are not mutually exclusive with neurophysiological accounts based on hemispheric asymmetry and locus coeruleus–autonomic regulation. Rather, they highlight the importance of cautious interpretation and motivate future studies employing longer recording durations, motion monitoring, and multimodal measurements to disentangle neural and non-neural contributions to variability-based pupillometric indices.

This study has several limitations that must be acknowledged. First, because of the practical constraints of working with young children, we could not use rigid head fixation methods, such as chin rests or head restraints, nor could we extend the duration of the experimental sessions. However, future studies could incorporate accelerometer sensors attached to the child's head to better detect and correct motion artifacts during postprocessing. Additionally, the use of more sophisticated and engaging attention-getting stimuli may allow for longer recording durations, enabling the application of advanced analytical metrics for pupil dynamics, which have been validated in previous studies (Nobukawa et al., 2021b,a; Kumano et al., 2022; Nobukawa et al., 2024). Second, establishing the test–retest reliability of VarLRdiff is an essential next step. Short-term repeated measurements, including same-day and different-day assessments, would clarify whether VarLRdiff primarily reflects stable individual traits or non-specific intra-individual variability, which has been widely emphasized as a core behavioral feature of ADHD (Gould et al., 2001; Kofler et al., 2013). Demonstrating adequate test–retest stability would substantially strengthen the interpretability and potential clinical utility of VarLRdiff. In addition, the present study did not assess personality traits or other attention- or executive function–related characteristics beyond ADHD-related tendencies. Therefore, we cannot exclude the possibility that VarLRdiff reflects broader individual differences in attentional or regulatory processes rather than ADHD-specific mechanisms. Third, according to the framework proposed by the U.S. Food and Drug Administration and the National Institutes of Health, a biomarker requires validation in terms of diagnostic specificity, reliability, and predictive utility (Califf, 2018). Because the present study is based on cross-sectional correlational analyses in a non-clinical sample, VarLRdiff should be interpreted as a candidate physiological correlate, rather than a validated clinical biomarker. Therefore, longitudinal studies are essential to determine whether VarLRdiff can serve as a reliable biomarker of ADHD. Tracking participants with high ADHD trait scores over time could provide valuable insights into the stability and predictive utility of this metric. These points should be carefully addressed in future studies to establish the validity and clinical utility of VarLRdiff as a candidate biomarker, pending further longitudinal and clinical validation.

In conclusion, we demonstrated that temporal variability in interocular pupil asymmetry (VarLRdiff) is significantly associated with ADHD-related traits in preschool and early school-aged children. This finding suggests that VarLRdiff may serve as a sensitive physiological marker reflecting the combined effects of immature autonomic regulation and neural asymmetry, both of which are characteristic of ADHD during the early developmental stages. Although other pupil-based indices such as the mean pupil size (LR) and temporal variability (VarLR) did not show significant associations with ADHD traits in this study, VarLRdiff emerged as a promising metric reflecting ADHD-related traits. Taken together, the present findings should be interpreted as exploratory and hypothesis-generating, based on the parallel evaluation of multiple pupil-derived features. Accordingly, further longitudinal and clinical studies with predefined primary outcomes are required to determine whether VarLRdiff can be validated as a reliable biomarker.

## Data Availability

The datasets presented in this article are not readily available because the informed consent did not include the declaration regarding publication of data. Requests to access the datasets should be directed to nobukawa@it-chiba.jp.

## References

[B1] American Psychiatric Association (2013). Diagnostic and Statistical Manual of Mental Disorders: DSM-5. Washington, DC: American Psychiatric Association. doi: 10.1176/appi.books.9780890425596

[B2] Artoni P. Piffer A. Vinci V. LeBlanc J. Nelson C. A. Hensch T. K. . (2019). Deep learning of spontaneous arousal fluctuations detects early cholinergic defects across neurodevelopmental mouse models and patients. Proc. Natl. Acad. Sci. U.S.A. 117, 23298–23303. doi: 10.1073/pnas.1820847116 31332003 PMC7519255

[B3] Aston-Jones G. Cohen J. D. (2005). An integrative theory of locus coeruleus-norepinephrine function: adaptive gain and optimal performance. Annu. Rev. Neurosci. 28, 403–450. doi: 10.1146/annurev.neuro.28.061604.135709 16022602

[B4] Balázs J. Keresztény Á. (2014). Subthreshold attention deficit hyperactivity in children and adolescents: a systematic review. Eur. Child Adolesc. Psychiatry 23, 393–408. doi: 10.1007/s00787-013-0514-7 24399038

[B5] Bouma H. Baghuis L. (1971). Hippus of the pupil: periods of slow oscillations of unknown origin. Vision Res. 11, 1345–1351. doi: 10.1016/0042-6989(71)90016-2 5148578

[B6] Bufo M. R. Guidotti M. De Faria C. Mofid Y. Bonnet-Brilhault F. Wardak C. . (2022). Autonomic tone in children and adults: pupillary, electrodermal and cardiac activity at rest. Int. J. Psychophysiol. 180, 68–78. doi: 10.1016/j.ijpsycho.2022.07.009 35914548

[B7] Califf R. M. (2018). Biomarker definitions and their applications. Exp. Biol. Med. 243, 213–221. doi: 10.1177/1535370217750088 29405771 PMC5813875

[B8] Castro Ribeiro T. García Pagès E. Huguet A. Alda J. A. Badiella L. Aguiló J. (2024). Physiological parameters to support attention deficit hyperactivity disorder diagnosis in children: a multiparametric approach. Front. Psychiatry 15:1430797. doi: 10.3389/fpsyt.2024.1430797 39575190 PMC11578978

[B9] Chen C. Lidstone D. Crocetti D. Mostofsky S. Nebel M. (2021). Increased interhemispheric somatomotor functional connectivity and mirror overflow in ADHD. NeuroImage 31:102759. doi: 10.1016/j.nicl.2021.102759 34280835 PMC8319349

[B10] Chen H. Yang Y. Odisho D. Wu S. Yi C. Oliver B. G. . (2023). Can biomarkers be used to diagnose attention deficit hyperactivity disorder? Front. Psychiatry 14:1026616. doi: 10.3389/fpsyt.2023.1026616 36970271 PMC10030688

[B11] Coghill D. Seth S. (2015). Effective management of attention-deficit/hyperactivity disorder (ADHD) through structured re-assessment: the Dundee ADHD clinical care pathway. Child Adolesc. Psychiatry Ment. Health 9, 1–14. doi: 10.1186/s13034-015-0083-2 26587055 PMC4652349

[B12] Curatolo P. D'Agati E. Moavero R. (2010). The neurobiological basis of ADHD. Ital. J. Pediatr. 36, 1–7. doi: 10.1186/1824-7288-36-79 21176172 PMC3016271

[B13] Doi H. Shinohara K. (2017). fNIRS studies on hemispheric asymmetry in atypical neural function in developmental disorders. Front. Hum. Neurosci. 11:137. doi: 10.3389/fnhum.2017.00137 28446869 PMC5388750

[B14] Dor-Ziderman Y. Zeev-Wolf M. Klein E. H. Bar-Oz D. Nitzan U. Maoz H. . (2021). High-gamma oscillations as neurocorrelates of ADHD: a MEG crossover placebo-controlled study. J. Psychiatr. Res. 137, 186–193. doi: 10.1016/j.jpsychires.2021.02.050 33684643

[B15] Douglas P. Gutman B. Anderson A. Larios C. Lawrence K. E. Narr K. . (2018). Hemispheric brain asymmetry differences in youths with attention-deficit/hyperactivity disorder. NeuroImage 18, 744–752. doi: 10.1016/j.nicl.2018.02.020 29876263 PMC5988460

[B16] DuPaul G. J. Power T. J. Anastopoulos A. D. Reid R. (2016). ADHD Rating Scale - *5 For Children and Adolescents: Checklists, Norms, and Clinical Interpretation*. New York, NY: Guilford Publications.

[B17] Faraone S. V. Banaschewski T. Coghill D. Zheng Y. Biederman J. Bellgrove M. A. . (2021). The world federation of ADHD international consensus statement: 208 evidence-based conclusions about the disorder. Neurosci. Biobehav. Rev. 128, 789–818. doi: 10.1016/j.neubiorev.2021.01.022 33549739 PMC8328933

[B18] Gould T. D. Bastain T. M. Israel M. E. Hommer D. W. Castellanos F. X. (2001). Altered performance on an ocular fixation task in attention-deficit/hyperactivity disorder. Biol. Psychiatry 50, 633–635. doi: 10.1016/S0006-3223(01)01095-2 11690600

[B19] Hayes T. R. Petrov A. A. (2016). Mapping and correcting the influence of gaze position on pupil size measurements. Behav. Res. Methods 48, 510–527. doi: 10.3758/s13428-015-0588-x 25953668 PMC4637269

[B20] He N. Palaniyappan L. Linli Z. Guo S. (2022). Abnormal hemispheric asymmetry of both brain function and structure in attention deficit/hyperactivity disorder: a meta-analysis of individual participant data. Brain Imaging Behav. 16, 54–68. doi: 10.1007/s11682-021-00476-x 34021487

[B21] Hooge I. T. Niehorster D. C. Hessels R. S. Cleveland D. Nyström M. (2021). The pupil-size artefact (PSA) across time, viewing direction, and different eye trackers. Behav. Res. Methods 53, 1986–2006. doi: 10.3758/s13428-020-01512-2 33709298 PMC8516786

[B22] Idrees I. Bellato A. Cortese S. Groom M. J. (2023). The effects of stimulant and non-stimulant medications on the autonomic nervous system (ANS) functioning in people with ADHD: a systematic review and meta-analysis. Neurosci. Biobehav. Rev. 144:104968. doi: 10.1016/j.neubiorev.2022.104968 36427764

[B23] Kofler M. J. Rapport M. D. Sarver D. E. Raiker J. S. Orban S. A. Friedman L. M. . (2013). Reaction time variability in ADHD: a meta-analytic review of 319 studies. Clin. Psychol. Rev. 33, 795–811. doi: 10.1016/j.cpr.2013.06.001 23872284

[B24] Kumano H. Nobukawa S. Shirama A. Takahashi T. Takeda T. Ohta H. . (2022). Asymmetric complexity in a pupil control model with laterally imbalanced neural activity in the locus coeruleus: a potential biomarker for attention-deficit/hyperactivity disorder. Neural Comput. 34, 2388–2407. doi: 10.1162/neco_a_01545 36283044

[B25] Lenartowicz A. Loo S. K. (2014). Use of EEG to diagnose ADHD. Curr. Psychiatry Rep. 16, 1–11. doi: 10.1007/s11920-014-0498-0 25234074 PMC4633088

[B26] Levantini V. Muratori P. Inguaggiato E. Masi G. Milone A. Valente E. . (2020). Eyes are the window to the mind: eye-tracking technology as a novel approach to study clinical characteristics of ADHD. Psychiatry Res. 290:113135. doi: 10.1016/j.psychres.2020.113135 32505031

[B27] Liu Y. Rodenkirch C. Moskowitz N. Schriver B. Wang Q. (2017). Dynamic lateralization of pupil dilation evoked by locus coeruleus activation results from sympathetic, not parasympathetic, contributions. Cell Rep. 20, 3099–3112. doi: 10.1016/j.celrep.2017.08.094 28954227 PMC5679481

[B28] MacDonald H. J. Kleppe R. Szigetvari P. D. Haavik J. (2024). The dopamine hypothesis for ADHD: an evaluation of evidence accumulated from human studies and animal models. Front. Psychiatry 15:1492126. doi: 10.3389/fpsyt.2024.1492126 39619336 PMC11604610

[B29] Maness E. B. Burk J. A. McKenna J. T. Schiffino F. L. Strecker R. E. McCoy J. G. . (2022). Role of the locus coeruleus and basal forebrain in arousal and attention. Brain Res. Bull. 188, 47–58. doi: 10.1016/j.brainresbull.2022.07.014 35878679 PMC9514025

[B30] McLaren J. W. Erie J. C. Brubaker R. F. (1992). Computerized analysis of pupillograms in studies of alertness. Invest. Ophthalmol. Vis. Sci. 33, 671–676. 1544791

[B31] McLennan J. D. (2016). Understanding attention deficit hyperactivity disorder as a continuum. Can. Fam. Physician 62, 979–982. 27965331 PMC5154646

[B32] Mehta T. R. Monegro A. Nene Y. Fayyaz M. Bollu P. C. (2019). Neurobiology of ADHD: a review. Curr. Dev. Disord. Rep. 6, 235–240. doi: 10.1007/s40474-019-00182-w

[B33] Musullulu H. (2025). Evaluating attention deficit and hyperactivity disorder (ADHD): a review of current methods and issues. Front. Psychol. 16:1466088. doi: 10.3389/fpsyg.2025.1466088 40066184 PMC11891363

[B34] Nobukawa S. Shirama A. Takahashi T. Takeda T. Ohta H. Kikuchi M. . (2021a). Identification of attention-deficit hyperactivity disorder based on the complexity and symmetricity of pupil diameter. Sci. Rep. 11:8439. doi: 10.1038/s41598-021-88191-x 33875772 PMC8055872

[B35] Nobukawa S. Shirama A. Takahashi T. Takeda T. Ohta H. Kikuchi M. . (2021b). Pupillometric complexity and symmetricity follow inverted-U curves against baseline diameter due to crossed locus coeruleus projections to the edinger-westphal nucleus. Front. Physiol. 12:92. doi: 10.3389/fphys.2021.614479 33643064 PMC7905168

[B36] Nobukawa S. Shirama A. Takahashi T. Toda S. (2024). Recent trends in multiple metrics and multimodal analysis for neural activity and pupillometry. Front. Neurol. 15:1489822. doi: 10.3389/fneur.2024.1489822 39687402 PMC11646859

[B37] Poynter W. D. (2017). Pupil-size asymmetry is a physiologic trait related to gender, attentional function, and personality. Laterality 22, 654–670. doi: 10.1080/1357650X.2016.1268147 27973982

[B38] Rocco I. Corso B. Bonati M. Minicuci N. (2021). Time of onset and/or diagnosis of ADHD in European children: a systematic review. BMC Psychiatry 21, 1–24. doi: 10.1186/s12888-021-03547-x 34784913 PMC8594188

[B39] Shirama A. Takeda T. Ohta H. Iwanami A. Toda S. Kato N. . (2020). Atypical alert state control in adult patients with ADHD: a pupillometry study. PLoS ONE 15:e0244662. doi: 10.1371/journal.pone.0244662 33378354 PMC7773233

[B40] Slater J. Joober R. Koborsy B. L. Mitchell S. Sahlas E. Palmer C. . (2022). Can electroencephalography (EEG) identify ADHD subtypes? A systematic review. Neurosci. Biobehav. Rev. 139:104752. doi: 10.1016/j.neubiorev.2022.104752 35760387

[B41] Socanski D. Aurlien D. Herigstad A. Thomsen P. H. Larsen T. K. (2013). Epilepsy in a large cohort of children diagnosed with attention deficit/hyperactivity disorders (ADHD). Seizure 22, 651–655. doi: 10.1016/j.seizure.2013.04.021 23711613

[B42] Sonuga-Barke E. J. Halperin J. M. (2010). Developmental phenotypes and causal pathways in attention deficit/hyperactivity disorder: potential targets for early intervention? J. Child Psychol. Psychiatry 51, 368–389. doi: 10.1111/j.1469-7610.2009.02195.x 20015192

[B43] Ukai K. Tsuchiya K. Ishikawa S. (1997). Induced pupillary hippus following near vision: increased occurrence in visual display unit workers. Ergonomics 40, 1201–1211. doi: 10.1080/001401397187441 9375534

[B44] Usui S. Stark L. (1982). A model for nonlinear stochastic behavior of the pupil. Biol. Cybern. 45, 13–21. doi: 10.1007/BF00387209 7126688

[B45] Wainstein G. Rojas-Líbano D. Crossley N. Carrasco X. Aboitiz F. Ossandón T. (2017). Pupil size tracks attentional performance in attention-deficit/hyperactivity disorder. Sci. Rep. 7:8228. doi: 10.1038/s41598-017-08246-w 28811624 PMC5557799

[B46] Winston M. Zhou A. Rand C. M. Dunne E. C. Warner J. J. Volpe L. J. . (2020). Pupillometry measures of autonomic nervous system regulation with advancing age in a healthy pediatric cohort. Clin. Auton. Res. 30, 43–51. doi: 10.1007/s10286-019-00639-3 31555934

[B47] Wolraich M. Brown L. Brown R. DuPaul G. Earls M. Feldman H. M. . (2011). ADHD: clinical practice guideline for the diagnosis, evaluation, and treatment of attention-deficit/hyperactivity disorder in children and adolescents. Pediatrics 128, 1007–1022. doi: 10.1542/peds.2011-2654 22003063 PMC4500647

[B48] Zhan C. Liu Y. Wu K. Gao Y. Li X. (2017). Structural and functional abnormalities in children with attention-deficit/hyperactivity disorder: a focus on subgenual anterior cingulate cortex. Brain Connect. 7, 106–114. doi: 10.1089/brain.2016.0444 28173729 PMC5359690

