# Supplementary material for: Temporal variability in pupillary asymmetry reflects ADHD-related traits in preschool and early school-aged children
Source: Front Cognit. 2026 Mar 11;5:1781233. doi: 10.3389/fcogn.2026.1781233 (PMC13281042; doi:10.3389/fcogn.2026.1781233)
Supplement: Supplementary file 1 [file Data_Sheet_1.PDF]

Supplementary Materials: Temporal Variability in Pupillary Asymmetry Reflects ADHD-Related Traits in Preschool and Early School-Aged Children

1 NUMBER OF VALID EPOCHS

In the pupillometry analysis, a total of 300 epochs were initially segmented for each participant. After excluding epochs containing excessive missing data or tracking loss, the number of valid epochs per participant ranged from 72 to 260. The mean ($\pm$ SD) number of valid epochs was 177.2 ± 34.6.

2 CHARACTERISTICS OF PUPIL METRICS

In the main text, pupil size (LR), temporal variability (VarLR), interocular asymmetry between the left and right pupils (LRdiff), and temporal variability of interocular asymmetry (VarLRdiff) were analyzed. Normality of these metrics was assessed using the Kolmogorov–Smirnov test. The results indicated that some metrics deviated from a normal distribution (LR: $p = 0.7166$, LRdiff: $p = 0.7850$, VarLR: $p = 0.0691$, VarLRdiff: $p = 0.0048$). Because VarLRdiff did not follow a normal distribution, Spearman's rank correlation coefficients (ρ) were used for correlation analyses throughout the study.

3 CORRELATION ANALYSIS OF MISSING RATES INVOLVING BLINK-RELATED CAUSES AND ADHD RATING SCALE–5 (ADHD-RS-5)

To evaluate whether missing values in pupil time series influenced the association between the temporal variability of interocular pupil asymmetry (VarLRdiff) and ADHD-related traits, we examined the relationship between missing data rates and ADHD Rating Scale–5 (ADHD-RS-5) percentile scores. Specifically, the proportion of missing samples was computed for each epoch using a 0.5-s window and then averaged across epochs for each participant. Spearman's rank correlation coefficients (ρ) were calculated between the average missing-value ratio and the total ADHD-RS-5 percentile score. No significant correlation was observed ($\rho = 0.14$, $p = 0.296$).

In addition, to assess the influence of linear interpolation on the main findings, VarLRdiff was recomputed without applying any interpolation to missing values in the pupil time series. Spearman's rank correlation coefficients between VarLRdiff and ADHD-RS-5 scores were then recalculated. Significant correlations were confirmed for the total score ($\rho = 0.35$, $p = 0.007$), inattentiveness (IN) subscale ($\rho = 0.40$, $p = 0.002$), and hyperactivity/impulsivity (Hyp/I) subscale ($\rho = 0.35$, $p = 0.007$), consistent with the results obtained using the interpolated data and reported in the main text.
